# LncRNA LOXL1-AS1 promotes esophageal squamous cell carcinoma progression by targeting DESC1

**DOI:** 10.7150/jca.51136

**Published:** 2021-01-01

**Authors:** Hongle Li, Jie Chu, Jinlin Jia, Jinxiu Sheng, Xue Zhao, Yurong Xing, Fucheng He

**Affiliations:** 1 Department of Molecular Pathology, The Affiliated Cancer Hospital of Zhengzhou University, Zhengzhou, Henan 450008, China.; 2 Department of Medical Laboratory, The First Affiliated Hospital of Zhengzhou University, Zhengzhou, Henan 450052, China.; 3 Institute of Medical and Pharmaceutical Science, Zhengzhou University, Zhengzhou, Henan 450052, China.; 4 Department of Physical Examination, The First Affiliated Hospital of Zhengzhou University, Zhengzhou, Henan 450052, China.

**Keywords:** Esophageal squamous cell carcinoma, long non-coding RNA, LOXL1-AS1, DESC1

## Abstract

Recently, ample evidence indicated that numerous aberrantly expressed long non-coding RNAs (lncRNAs) participated in the development of multiple malignancies. However, the expression and function of lncRNA LOXL1-AS1 in mediating esophageal squamous cell carcinoma (ESCC) carcinogenesis remains largely elusive. Here we validated that LOXL1-AS1 was significantly upregulated in ESCC tissues compared with the corresponding adjacent non-neoplastic tissues, and LOXL1-AS1 expression was positively correlated with ESCC patients' lymph node metastasis. Besides, LOXL1-AS1 knockdown impaired ESCC cells proliferation, migration and invasion capabilities *in vitro*. Furthermore, inhibiting LOXL1-AS1 in ESCC cells increased the percentage of cells at the G1 phase, accompanied by reducing in S phase in contrast to scramble control, and silencing of LOXL1-AS1 evoked ESCC cell apoptosis. From high throughput RNA sequencing (RNA-seq) analysis, we identified that differentially expressed in squamous cell carcinoma 1 (DESC1) was a critical downstream target of LOXL1-AS1. Taken together, we demonstrated the function and mechanism of LOXL1-AS1 in contributing ESCC progression for the first time, and indicated LOXL1-AS1 may be a novel therapeutic biomarker of ESCC.

## Introduction

Esophageal cancer (EC) is one of the most leading reasons of cancer-related death worldwide 1,2. Esophageal cancer contains two major subtypes, including esophageal adenocarcinoma (EAC) and esophageal squamous cell carcinoma (ESCC) 3. EC has becoming a global serious problem due to threatening life, and ESCC patients with advanced stage tumors commonly harbor poor prognosis 4. Despite surgery is the traditional treatment for ESCC patients, and developing new therapeutic strategies for ESCC is of great significance. Thus, understanding the molecular mechanisms underlying ESCC progression would contribute to identify new diagnostic and therapeutic targets.

Long noncoding RNAs (lncRNAs) are described as regulatory RNA transcripts, characterized by more than 200 nucleotides in length that are often lacking in protein-coding ability 5. Emerging studies have found numerous lncRNAs involved in the occurrence and development of many malignant tumors 6-8. For example, lncRNA DLEU2 accelerated hepatocellular carcinoma (HCC) cells proliferative, migratory and invasive abilities by binding EZH2 to aggravate the progression of HCC 9. In addition, lncRNAs function as regulatory RNA molecules in cancer biological processes, which can regulate cell proliferation, apoptosis, cell cycle, metastasis and drug resistance 10,11. Furthermore, accumulating evidence indicated that dysregulated lncRNAs could act as tumor suppressors or oncogenes to participate in cancer progression 12,13. For example, lncRNA FENDRR was downregulated in NSCLC tissues, and suppressed the progression of non-small cell lung cancer (NSCLC) via binding to miR-761 and regulating TIMP2 expression 14. Additionally, knockdown lncRNA FOXD3-AS1 inhibited cutaneous malignant melanoma cells proliferation, invasion and migration via regulating miR-325/MAP3K2 axis 15.

LncRNA LOXL1 antisense RNA 1 (LOXL1-AS1) is an antisense lncRNA, which is located at human chromosome 15 (q24.1) with 10781 bp 16. The biological function of LOXL1-AS1 has been explored in various cancers, including osteosarcoma, prostate cancer, gastric cancer, glioblastoma, and breast cancer 17-21. Furthermore, LOXL1-AS1 can regulate tumor occurrence, development, and metastasis through various mechanisms as a novel regulator of tumorigenesis 22. For example, knockdown of LOXL1-AS1 dramatically inhibited osteosarcoma cell proliferation, migration and invasion through suppressing PI3K-AKT pathway 17. LOXL1-AS1 contributed to aggressive biological processes related with mesenchymal subtype via NF-κB signaling 20.

In this study, we elucidated that LOXL1-AS1 was evidently upregulated in ESCC tissues and high expression of LOXL1-AS1 was associated with ESCC lymph node metastasis. Furthermore, we confirmed that LOXL1-AS1 enhances ESCC cell proliferation, migration, and invasion capacities, and LOXL1-AS1 regulates ESCC cell cycle and apoptosis progression. Besides, LOXL1-AS1 promotes ESCC progression by targeting DESC1, indicating its critical role in ESCC progression.

## Materials and methods

### Tissue samples and clinical data collection

Forty-five pairs of ESCC fresh tissues and matched adjacent non-tumorous tissues were collected from patients with esophagectomy in the First Affiliated Hospital of Zhengzhou University. None of these ESCC patients received chemotherapy or radiotherapy before surgery, and all patients were finally diagnosed by histopathology at the Department of Thoracic Surgery. The procedures were conducted in accordance with the Declaration of Helsinki, and tissue samples were stored in liquid nitrogen upon receipt for further study. Written informed consents were obtained from all patients before the operation, and we collected the clinicopathological features of these patients from electronic medical records. The study was approved by the Ethics Committee of the First Affiliated Hospital of Zhengzhou University.

### Cell culture

The human ESCC cell lines KYSE30 and EC109 were purchased from Shanghai Institute of Life Sciences cell bank center (Shanghai, China). The cell lines were cultured in Roswell Park Memorial Institute-1640 medium (RPMI-1640, Hyclone, UT, USA) supplemented with 10% fetal bovine serum (FBS, Gibco, USA), maintained in 5% CO_2_ incubator under 37 °C and stored in CELLSAVING (New Cell & Molecular Biotech, Suzhou, China) at -80 °C. All cell culture dishes were purchased from Hangzhou Xinyou Biotechnology Co., Ltd.

### RNA isolation and quantitative real-time PCR

Total RNAs from frozen ESCC tissues and cell lines were extracted by TriZol reagent (TakaRa, Dalian, China) and subjected to reverse-transcribed reaction using the PrimeScript™RT reagent Kit with gDNA Eraser (TakaRa, Dalian, China) according to the manufacturer's instructions. The mRNA levels of specific genes were measured by using the kit specification of SYBR® Premix Ex Taq™ II (TakaRa, Dalian, China) on LightCycler 480 II Real-Time PCR System (Roche, Basel, Switzerland). The relative expression levels of mRNAs were normalized to GAPDH. The primers used in the current study were shown as follows: LOXL1-AS1: 5'-TTCCCATTTACCTGCCCGAAG-3' (forward), 5'-GTCAGCAAACACATGGCAAC-3' (reverse); miR-34a: 5'-CCCACATTTCCTTCTTATCAACAG-3' (forward), 5'-GGCATCTCTCGCTTCATCTT-3' (reverse); DESC1: 5'-ACAGGATTTGGAGCACTGA-3' (forward), 5'-GCCAGCACATAACATTCTAGG-3' (reverse); GAPDH: 5'-GGAGCGAGATCCCTCCAAAAT-3' (forward), 5'-GGCTGTTGTCATACTTCTCATGG-3' (reverse); U6: 5'-CGCTTCGGCAGCACATATAC-3' (forward), 5'-CAGGGGCCATGCTAATCTT-3' (reverse).

### RNA interference

Small interfering RNA of LOXL1-AS1 (siLOXL1-AS1), and scramble siRNA of LOXL1-AS1 (siRNA control) were synthesized by RiboBio Co., Ltd (Guangzhou, China), and transfected using RFect siRNA Transfection Reagent (Changzhou Biogenerating Biotechnologies Co., Ltd) according to the manufacturer's protocol. RT-PCR was conducted to detect gene silence efficiency.

### *In vitro* migration and invasion assays

Transwell assays were conducted to determine the cell motility using the transwell 24-well chambers with or without Matrigel as described before 23. Transfected cells were collected and resuspended in serum-free RPMI-1640 medium. 5 × 10^4^ cells in 200 μl serum-free medium were placed into the upper chamber while complete medium containing 15% FBS was added into the lower chamber. After incubation at 37 °C for 24 h, the cells on lower membrane surface were fixed by 4% paraformaldehyde and stained by crystal violet. We randomly selected five fields of vision from each chamber under the microscope for cell counting to assess the abilities of cell migration and invasion.

### Cell proliferation assay

The cell viability was performed using the WST-1 Assay Kit (Beyotime Biotechnology, Shanghai, China) as previously described 24. Briefly, CRC cells transfected with siRNA were seeded in 96-well plates at the density of 2 × 10^3^. After incubating for 24, 48, 72, 96 hours, 10 μl WST-1 reagent (Roche) was added into the relevant cells. SpctraMax M5 (Molecular Devices, San Francisco, CA, USA) was utilized to measure the cell proliferation rates.

### Apoptosis assay

For apoptosis analysis, ESCC cells transfected with siRNA were harvested after 48 h. According to manufacturer's instructions, the transfected cells were treated with fluorescein isothiocyanate (FITC)-Annexin V and propidium iodide (PI) for 30 min at room temperature off from the light. Subsequently, the cell apoptosis were analyzed by flow cytometry (FACScan; BD Biosciences, Shanghai, China).

### Cell cycle assay

For cell cycle distribution, the transfected cells were harvested and washed twice with cold PBS. Then cells were resuspended and fixed in 70% pre-cooled ethanol at 4 °C overnight. On the next day, cells were washed by PBS and treated with PI for 30 min away from light at room temperature. We used flow cytometry (FACScan; BD Biosciences, Shanghai, China) to examine the percentages of cells in G0/G1, S and G2/M phase.

### Statistical analysis

Statistical evaluations were performed with the SPSS 21.0 software (SPSS Inc., Chicago, IL, USA) and GraphPad Prism 5 (GraphPad Software Inc., La Jolla, CA, USA). The experiments were repeated at least three times and experimental data were shown as the means ± standard deviation (SD). Student's *t*-test was carried out to compare between two groups. For category variable differences, Chi-square tests were performed. Comparison of multiple groups was analyzed via one-way analysis of variance (ANOVA). *P* < 0.05 was considered to be statistically significant.

## Results

### Elevated expression of lncRNA LOXL1-AS1 in ESCC tissues

**Figure 1:**
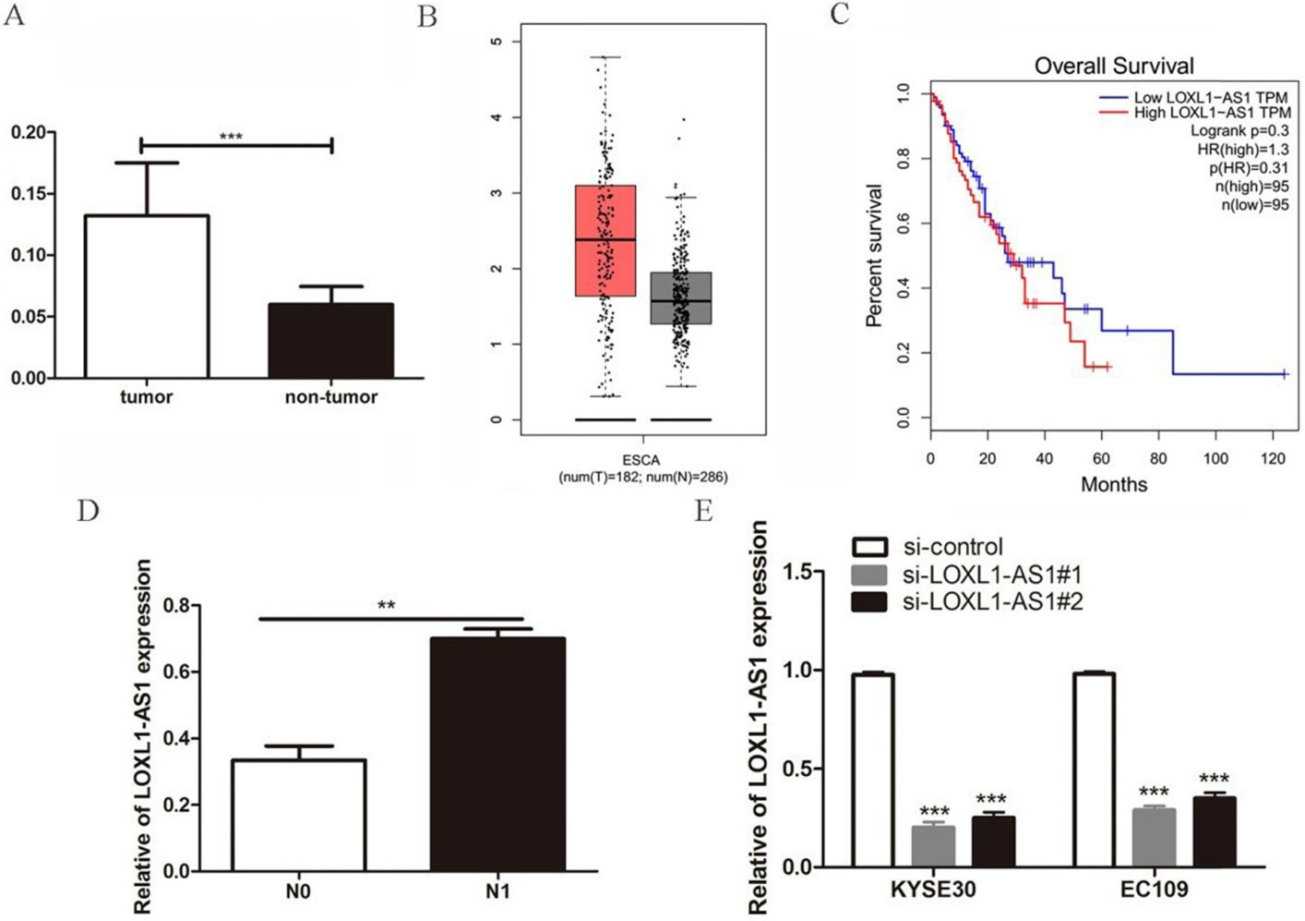
** Expression of the lncRNA LOXL1-AS1 was upregulated in ESCC tissues. (A)** The expression of LOXL1-AS1 in 45 paired ESCC tumor tissues and corresponding adjacent non-tumoral tissues. **(B)** The level of LOXL1-AS1 in human esophageal carcinoma (ESCA) and normal esophageal tissues from GEPIA database. **(C)** The Kaplan-Meier plot of LOXL1-AS1 expression in ESCA from GEPIA database. **(D)** Relative expression of LOXL1-AS1 in patients with lymph node metastasis (N1) compared with patients without lymph node metastasis (N0). **(E)** Two different siRNAs targeting LOXL1-AS1 were transfected in ESCC cells and RT-PCR was utilized to verify the knockdown efficiencies. **P < 0.01, ***P < 0.001.

To determine the role of LOXL1-AS1 in ESCC tissues, we first evaluated the expression of LOXL1-AS1 in 45 paired human ESCC tissues and corresponding adjacent non-neoplastic tissues by qRT-PCR experiment. We observed that LOXL1-AS1 was remarkably upregulated in ESCC tissues compared with the matched adjacent non-neoplastic tissues (Fig. 1A). Afterwards, accordant results were acquired by analyzing LOXL1-AS1 expression in esophageal carcinoma using the Gene Expression Profiling Interactive Analysis (GEPIA) database (http://gepia.cancer-pku.cn/) (Fig. 1B). In addition, the relationship between LOXL1-AS1 expression and the survival prognosis of ESCC patients was analyzed. Although the statistical significance was not obvious, the results showed that patients with high LOXL1-AS1 expression had worse prognosis compared with patients with low MNX1-AS1 expression (Fig. 1C).

Next, to further analyze the relationship between LOXL1-AS1 expression and the clinicopathological features of ESCC patients, the enrolled patients were divided into two groups on the basis of LOXL1-AS1 expression. The results indicated that LOXL1-AS1 expression was strongly correlated with the lymph node metastasis of ESCC patients (Table 1). The expression level of LOXL1-AS1 was evidently increased in ESCC samples with lymph node metastasis compared to those without metastasis (Fig. 1D). However, no correlation was discovered between LOXL1-AS1 expression and patients' age, gender, tumor size, differentiation grade or TNM stage. Taken together, these results indicate that LOXL1-AS1 is closely linked to ESCC carcinogenesis.

### LOXL1-AS1 knockdown inhibits ESCC cells proliferation, migration and invasion

**Figure 2:**
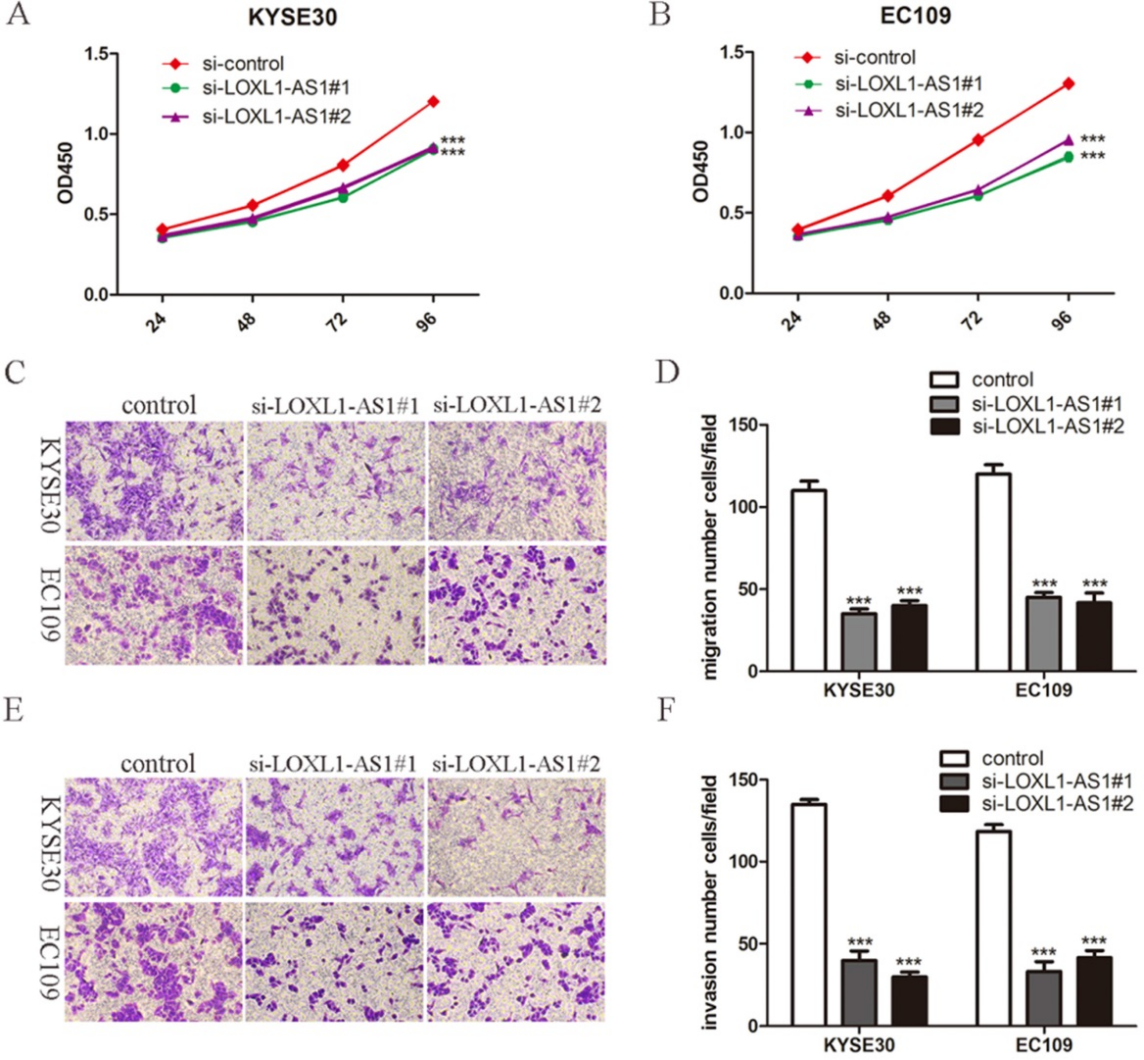
**LOXL1-AS1 knockdown inhibited ESCC cells proliferation, migration and invasion. (A-B)** The effects of LOXL1-AS1 on cell proliferation was performed using WST-1 assay. **(C-F)** Transwell assays were performed to determine the role of LOXL1-AS1 in cell migration and invasion of ESCC cells. Representative images of cell migration and invasion and statistical analysis of specific migrated or invaded cell number were shown. ***P < 0.001.

Given the high expression of LOXL1-AS1 was correlated with the lymph node metastasis in ESCC tissues, we next explored the biological function of LOXL1-AS1 in ESCC cells. KYSE30 and EC109 cells were transfected with siRNAs targeting LOXL1-AS1 to diminish the expression of LOXL1-AS1. LOXL1-AS1 was obviously inhibited in ESCC cells, as evidenced by RT-PCR analysis (Fig. 1E). We next performed WST-1 assays to detect the effects of LOXL1-AS1 on cell proliferation. The results showed that knockdown of LOXL1-AS1 could efficiently inhibit ESCC cell proliferation (Fig. 2A, B). Thus, the data revealed that LOXL1-AS1 regulated ESCC cells proliferation *in vitro*.

Subsequently, we investigated whether LOXL1-AS1 could influence the capabilities of ESCC cell migration and invasion. To this end, transwell assays were carried out and we found ESCC cell migration and invasion abilities were decreased when knocking down of LOXL1-AS1 in KYSE30 and EC109 cells (Fig. 2C-F). These findings suggested that LOXL1-AS1 significantly affected the ESCC cells migration and invasion.

### Knockdown of LOXL1-AS1 elicites cell cycle arrest and cell apoptosis

**Figure 3:**
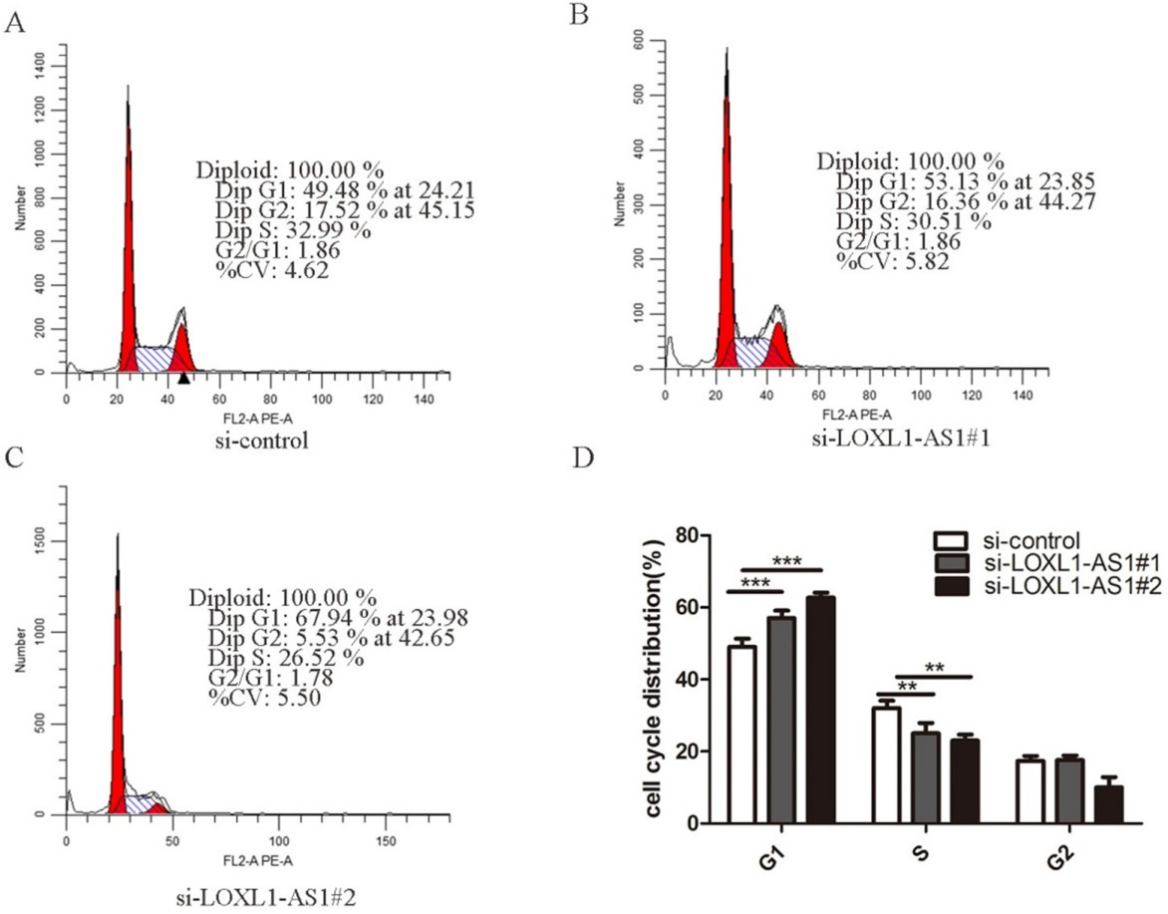
** LOXL1-AS1 regulated ESCC cell cycle progression. (A-C)** Flow cytometry assay was performed to analyze the cell cycle distribution of LOXL1-AS1 in KYSE30 cells. **(D)** The assays were repeated three times, and data were shown as mean ± SD from three independent experiments. **P < 0.01, *** *P* < 0.001.

**Figure 4:**
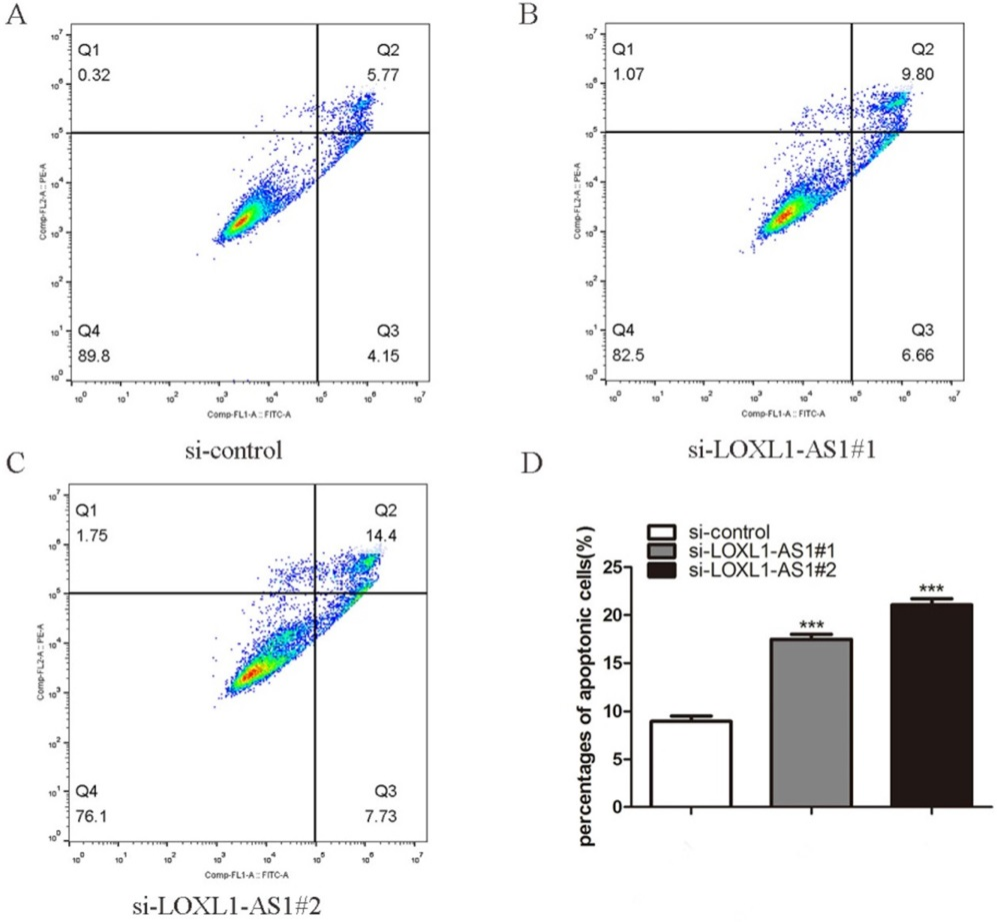
** LOXL1-AS1 deficiency promoted cellular apoptosis. (A-C)** The KYSE30 cells were transfected with LOXL1-AS1 siRNAs, and the cell apoptotic percentage of different group cells were examined. **(D)** The experiment was repeat triple, and data was exhibited from three independent experiments using statistical analysis. ***P < 0.001.

Next, flow cytometry analysis was performed to figure out whether LOXL1-AS1 affected the proliferation of ESCC cells by altering cell cycle distribution. The results manifested that inhibiting LOXL1-AS1 in KYSE30 cells increased the percentage of cells at the G1 phase, accompanied by reducing in S phase in contrast to scramble control (Fig. 3A-D). In addition, we analyzed the effect of LOXL1-AS1 on cell apoptosis. Using the Annexin V/PI double-staining method to measure the apoptotic percentage, flow cytometry assays demonstrated that silencing of LOXL1-AS1 induced ESCC cell apoptosis (Fig. 4A-D). Collectively, these findings suggested the pivotal role of LOXL1-AS1 in controlling ESCC cell cycle progression and apoptosis.

### DESC1 is a crucial downstream target of LOXL1-AS1

**Figure 5:**
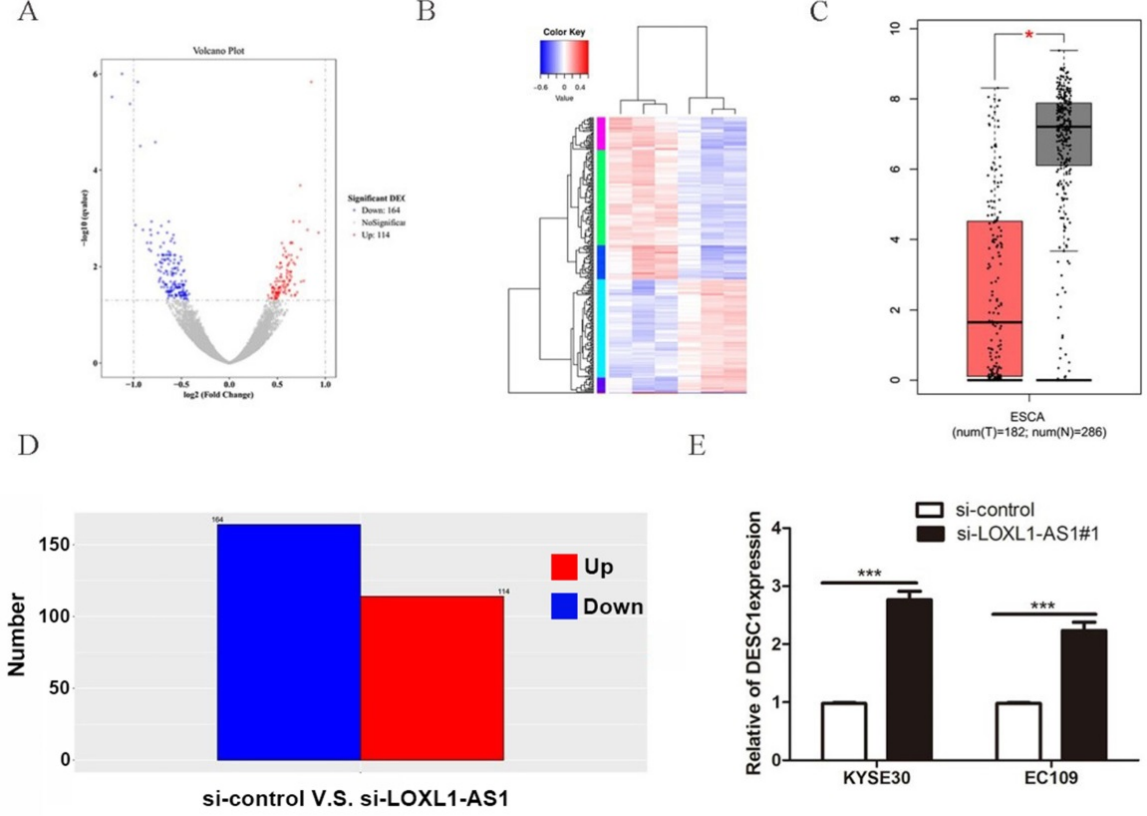
** DESC1 is a key downstream target of LOXL1-AS1. (A, D)** Screening of differentially expressed genes by RNA-seq (≥ 2-fold change) after knockdown of LOXL1-AS1 in KYSE30 cells through volcano plot **(A)** and bar chart **(D)**. **(B)** Hierarchical clustering of altered differential genes after inhibition of LOXL1-AS1 in KYSE30 cells. **(C)** The relative expression of DESC1 in human esophageal carcinoma (ESCA) compared with normal esophageal tissues in GEPIA database. **(E)** The expression of DESC1 was measured by qRT-PCR after silencing of LOXL1-AS1 in KYSE30 cells. *P <0.5, ***P < 0.001.

**Figure 6:**
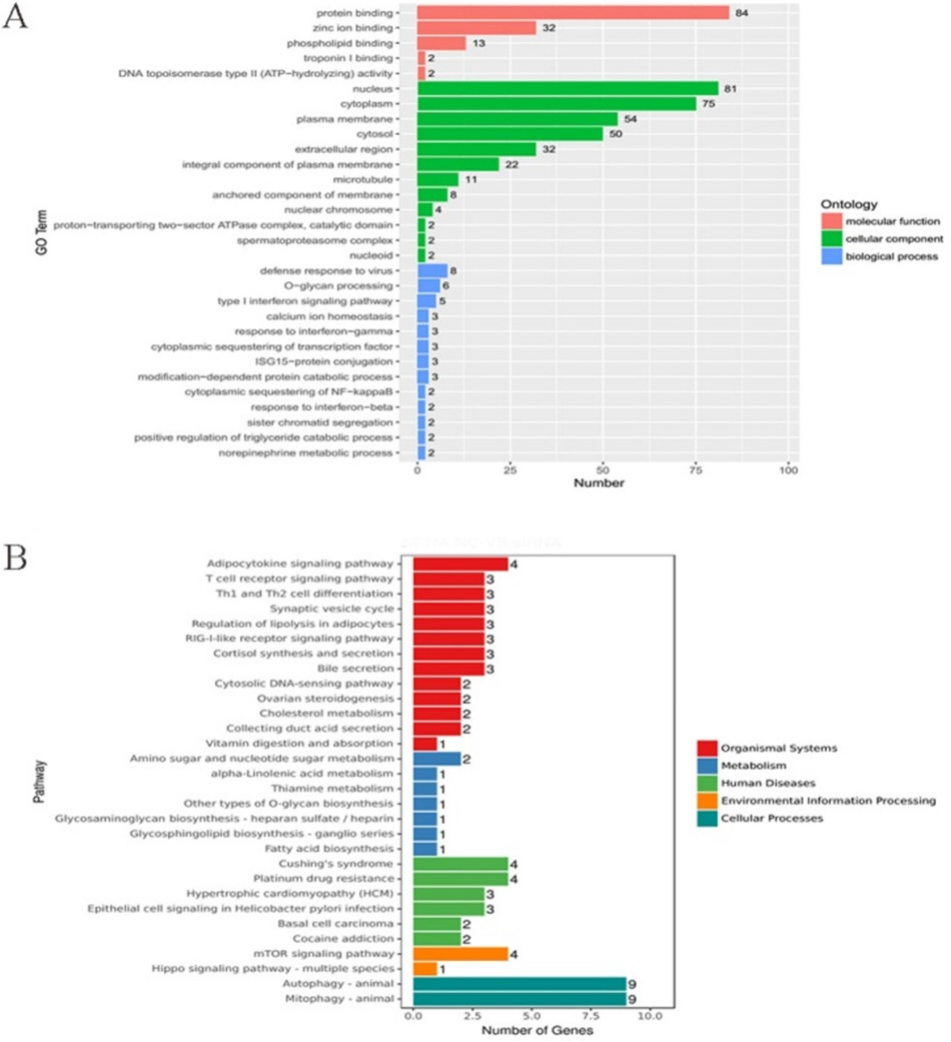
** GO and KEGG enrichment analysis of differentially expressed genes (DEGs) upon LOXL1-AS1 inhibition. (A-B)** Gene ontology (GO) and Kyoto Encyclopedia of Genes and Genomes (KEGG) enrichment analysis were performed on these DEGs.

To further explore the mechanisms of LOXL1-AS1 underlying ESCC progression, we performed transcriptome RNA-sequencing using cell lysates isolated from control and LOXL1-AS1 knockdown ESCC cells (si-control and si-LOXL1-AS1#1). Through the screening of differentially expressed genes (DEGs, ∣Log_2_FC∣ > 1 and the adjusted *P* value < 0.05), we found 278 differentially expressed mRNAs after depletion of LOXL1-AS1 in KYSE30 cells, including 164 upregulated and 114 downregulated genes (Fig. 5A, D). The hierarchical clustering showed that differentially expressed mRNAs were apparently distinguished between control cells and the LOXL1-AS1 knockdown cells (Fig. 5B). Next, Gene ontology (GO) and Kyoto Encyclopedia of Genes and Genomes (KEGG) enrichment analysis were conducted to investigate these differentially expressed genes. The enrichment analysis of GO biological process revealed that the changed molecular functions were mainly involved in a variety of processes, including protein binding and zinc ion binding (Fig. 6A). In addition, KEGG enrichment analysis indicated that these DEGs were closely relevant to various pathways, including autophagy, mitophagy, mTOR signaling pathway and adipocytokine signaling pathway (Fig. 6B).

Next, among these top DEGs, we selected differentially expressed in squamous cell carcinoma 1 (DESC1) for further analysis. DESC1 has been widely regarded as a tumor suppressor in multiple cancers. The expression of DESC1 was examined in GEPIA database, and the relative mRNA level of DESC1 was obviously downregulated in esophageal cancer tissues compared with adjacent normal tissues (Fig. 5C). Moreover, we performed qRT-PCR to measure the expression of DESC1 after silencing of LOXL1-AS1 and observed that DESC1 was markedly elevated in ESCC cells (Fig. 5E). These data exhibited that DESC1 was regulated by LOXL1-AS1 and may function as a novel downstream target of LOXL1-AS1.

## Discussion

Accumulating evidence manifested that lncRNAs have turned into important players during cellular development and human diseases, particularly in cancers 25. For example, lncRNA MIR31HG regulated cell cycle progression via HIF1A and p21 and facilitated head and neck cancer cell proliferation and tumorigenesis 26. LncRNA CDKN2BAS promoted hepatocellular carcinoma metastasis by regulating the miR-153-5p/ARHGAP18 signaling and predicted the poor prognosis in HCC patients 27. Most of the lncRNAs concerning the potential biological functions and underlying mechanistic details in human ESCC remain poorly understood. Esophageal squamous cell carcinoma is one of the most invasive cancers with an increasing incidence worldwide in recent years 28. Despite extensive clinical studies and encouraging progress has been made in the treatment of ESCC, the overall five-year survival for ESCC patients still remains dismal, owing to the propensity to invade nearby organs and distant metastasis 29. Therefore, there is an urgent need for understanding the molecular and biochemical mechanisms of advanced ESCC and to develop the novel therapeutic approaches for ESCC patients.

LncRNA LOXL1 antisense RNA 1 (LOXL1‐AS1) has reported to be an oncogenic modulator in multiple cancers 30. For example, LOXL1-AS1 acted as a ceRNA to upregulate USF1 via sponging miR-708-5p and facilitated the tumorigenesis and stemness of gastric carcinoma 19. In our study, we found LOXL1-AS1 was significantly upregulated in ESCC tissues compared with the corresponding adjacent non-neoplastic tissues. In addition, LOXL1-AS1 expression was tightly correlated with lymph node metastasis. Silencing of LOXL1-AS1 apparently inhibited ESCC cells proliferation, migration and invasion capabilities. Furthermore, inhibiting LOXL1-AS1 in ESCC cells increased the percentage of cells at the G1 phase, accompanied by reducing in S phase in contrast to scramble control. Meanwhile, silencing of LOXL1-AS1 accelerated ESCC cell apoptosis. To the best of our knowledge, this is the first study to delineate the functional role of LOXL1-AS1 in ESCC progression.

LOXL1-AS1 was previously reported to promote the medulloblastoma proliferation and metastasis through activating the PI3K-AKT pathway 31. LOXL1-AS1 could function as a ceRNA for miR-324-3p to contribute to cholangiocarcinoma progression via modulation of ATP-binding cassette transporter A1 32. However, the mechanism underlying LOXL1-AS1 in ESCC progression remains unknown. Herein, we performed RNA-seq to determine the possible downstream target genes of LOXL1-AS1. We found 164 upregulated mRNAs and 114 downregulated mRNAs after inhibition of LOXL1-AS1. Moreover, GO and KEGG analysis demonstrated these genes were mainly involved in diverse cancer-related signaling pathways. We selected the tumor-related gene DESC1 in our study. DESC1, which is also known as transmembrane protease serine 11E (TMPRSS11E), belongs to one of the members of type II transmembrane serineprotease (TTSP) family 33. Recent studies indicated DESC1 could behave as a tumor suppressor in multiple malignancies including ESCC 34. Moreover, GEPIA database manifested that the relative mRNA level of DESC1 was obviously downregulated in esophageal cancer tissues compared with adjacent normal tissues. qRT-PCR was performed to measure the expression of DESC1 after knockdown LOXL1-AS1 in ESCC cells and we observed that DESC1 was evidently upregulated. These findings indicated DESC1 is an important downstream effector of LOXL1-AS1 in regulating ESCC progression.

In summary, we have elucidated that LOXL1-AS1 may function as an oncogenic factor during ESCC development. Furthermore, LOXL1-AS1 expression was elevated in ESCC patients, and LOXL1-AS1 promoted ESCC progression by regulating DESC1 expression. Our data may provide evidence that LOXL1-AS1 served as a promising diagnostic biomarker and therapeutic target of ESCC.

## Figures and Tables

**Table 1:** The correlation between LOXL1-AS1 expression and clinicopathological factors of ESCC patients

Variables	Number	LOXL1-AS1 expression		*P*-value
		High group	Low group	
**Gender**				
Male	23	14	9	0.463
Female	22	11	11	
**Age**				
<65	19	12	7	0.712
≥65	26	15	11	
**Tumor size**				
≤3 cm	19	7	12	0.102
>3 cm	26	16	10	
**Differentiation grade**				
Poor	12	7	5	0.559
Well/Moderate	33	16	17	
**TNM stage**				
I+II	13	8	5	0.141
III+IV	32	12	20	
**Lymphatic metastasis**				
Negative	23	16	7	0.005**
Positive	22	6	16	

**P < 0.01.
